# Two Small Molecules Block Oral Epithelial Cell Invasion by *Porphyromons gingivalis*

**DOI:** 10.1371/journal.pone.0149618

**Published:** 2016-02-19

**Authors:** Meng-Hsuan Ho, Li Huang, J. Shawn Goodwin, Xinhong Dong, Chin-Ho Chen, Hua Xie

**Affiliations:** 1 School of Dentistry, Meharry Medical College, Nashville, Tennessee, United States of America; 2 Department of Surgery, Duke University Medical Center, Durham, North Carolina, United States of America; 3 Department of Biochemistry and Cancer Biology, Meharry Medical College, Nashville, Tennessee, United States of America; 4 Department of Microbiology, Meharry Medical College, Nashville, Tennessee, United States of America; Oregon Health & Science University, UNITED STATES

## Abstract

*Porphyromonas gingivalis* is a keystone pathogen of periodontitis. One of its bacterial characteristics is the ability to invade various host cells, including nonphagocytic epithelial cells and fibroblasts, which is known to facilitate *P*. *gingivalis* adaptation and survival in the gingival environment. In this study, we investigated two small compounds, Alop1 and dynasore, for their role in inhibition of *P*. *gingivalis* invasion. Using confocal microscopy, we showed that these two compounds significantly reduced invasion of *P*. *gingivalis* and its outer membrane vesicles into human oral keratinocytes in a dose-dependent manner. The inhibitory effects of dynasore, a dynamin inhibitor, on the bacterial entry is consistent with the notion that *P*. *gingivalis* invasion is mediated by a clathrin-mediated endocytic machinery. We also observed that microtubule arrangement, but not actin, was altered in the host cells treated with Alop1 or dynasore, suggesting an involvement of microtubule in this inhibitory activity. This work provides an opportunity to develop compounds against *P*. *gingivalis* infection.

## Introduction

*Porphyromonas gingivalis* is a gram-negative bacterium strongly associated with chronic periodontitis [1–3]. Recently, a keystone pathogen hypothesis regarding the pathogenesis of periodontitis was proposed, suggesting that the presence of *P*. *gingivalis* in the oral cavity, even at low levels, is capable of disturbing host–microbial homeostasis and inducing periodontitis [3,4]. The pathogenicity of *P*. *gingivalis* has been extensively characterized, including its abilities to colonize the surfaces of oral tissues, interact with other oral bacteria, induce a destructive immune response, and invade host cells [5–8]. All of these virulence features have been attractive therapeutic targets for preventing *P*. *gingivalis* infection.

Cell invasion by *P*. *gingivalis* is found in oral epithelial cells, gingival fibroblasts, aortic and heart endothelial cells, and vascular smooth muscle cells [9–12]. More significantly, *P*. *gingivalis*, once internalized, can multiply and persist within host cells [13,14]. *P*. *gingivalis* invasion is believed to protect the bacteria against environmental challenges, including innate immune surveillance systems and antibiotic treatment [15], which likely plays a pivotal role in chronic bacterial infection. The ability of *P*. *gingivalis* to invade host cells also appears to be critical in the progression of atherosclerosis [16]. Recently, we demonstrated that the outer membrane vesicles of *P*. *gingivalis* are also invasive and exhibit significantly higher invasion efficiency than their parental bacterial cells [17,18]. Studies of *P*. *gingivalis* invasion have provided insight into the mechanisms by which this organism invades nonphagocytic cells such as epithelial cells and fibroblasts. A number of bacterial proteins have been identified as ligands that interact with host receptors to initiate an internalization process. One of best known ligand/receptor interactions is the pair of FimA, a structural protein of the bacterial major fimbriae, and α5β1 integrin on the surface of epithelial cells [11,19]. The consequence of the specific ligand/receptor recognition results in cytoskeletal remodeling, which promotes the engulfment of bacteria [11,20]. Involvement of the cytoskeleton in *P*. *gingivalis* invasion is further supported by evidence that cytochalasin D, an inhibitor of actin polymerization, and nocodazole, an inhibitor of microtubule formation, inhibited *P*. *gingivalis* invasion of epithelial cells [21]. However, the mechanism of actin and microtubule in the bacterial invasion is not clear.

Since control of *P*. *gingivalis* infection by targeting bacterial invasion activity is limited, we attempted to identify inhibitory agents able to block *P*. *gingivalis* invasion. Building on our previous work identifying natural products as a new class of anti-influenza A virus agents [22], we focused on a natural lupine alkaloid, aloperine (Alop1), which is a known principal constituent of Sophora species used in traditional Chinese medicine against a variety of ailments [23,24]. Recently, we demonstrated that Alop1 and its derivatives were effective against the H1N1 influenza A virus, although the mechanism of Alop1’s action remains to be determined [22]. Based on the well-known fact that viral entry is involved in receptor-mediated endocytosis [25], we propose that Alop1 may also block the entry of *P*. *gingivalis* and its outer membrane vesicles (OMV) into primary oral keratinocytes. Another interesting endocytosis inhibitor is dynasore, a small compound first discovered by Macia *et al*. [26]. It is well-defined that dynasore specifically inhibits dynamin-mediated clathrin-coated vesicle formation during endocytosis. Our results revealed potent inhibitory activities of Alop1 and dynasore against invasion of *P*. *gingivalis* and its OMVs. We observed differential microtubule rearrangements in oral epithelial cells induced by Alop1 and dynasore, which may precede microtubule-dependent internalization and intracellular trafficking of *P*. *gingivalis*. These findings represent opportunities to use these two compounds as chemical probes for further characterization of *P*. *gingivalis* invasion and as leading compounds for drug development against *P*. *gingivalis* infection.

## Materials and Methods

### Bacterial strains and vesicle preparation and quantification

*P*. *gingivalis* ATCC 33277 was grown from frozen stocks in trypticase soy broth (TSB) or on TSB blood agar plates supplemented with yeast extract (1mg/ml), hemin (5 μg /ml), and menadione (1 μg/ml), and incubated at 37°C in an anaerobic chamber (85% N_2_, 10% H_2_, 5% CO_2_). *P*. *gingivalis* vesicles were prepared as previously described [27]. Briefly, *P*. *gingivalis* was grown to the late exponential phase and growth media were collected by centrifugation at 10,000 × *g* for 15 min at 4°C and filtered through a 0.22 μm pore size filter (CellTreat) to remove residual bacteria. Vesicles were collected by ultracentrifugation at 126,000 × *g* for 2 h at 4°C and resuspended in phosphate-buffered saline (PBS) containing 10% glycerol. Since quantifying bacterial vesicles by their protein or lipid content in weight represents the most common way to normalize data [28], proteins were extracted from vesicles using a BugBuster® Protein Extraction Reagent (Novagen). Protein concentrations were determined with a Bio-Rad Protein Assay Kit (Bio-Rad). To determine lipid content, *P*. *gingivalis* vesicles were resuspended in 100 μl sterile PBS and quantized using the fluorescent lipophilic dye FM4-64 (Molecular Probes). Fluorescence was measured at 506 nm (excitation) and 750 nm (emission) to obtain relative fluorescence units/ml [29].

### Preparation of compounds

Aloperine and nocodazole were purchased from Sigma-Aldrich. Alop1 denotes HPLC purified aloperine to ensure the purity of the compound is greater than 95%. Dynasore was purchased from Tocris Bioscience. Cytochalasin D was purchased from Invitrogen.

### Treatment of host cells with *P*. *gingivalis* 33277 and its vesicles

Human oral keratinocytes (HOKs) were purchased from ScienCell Research Laboratories (Carlsbad, CA) and cultured in specific media, according to the manufacturer’s instructions. Prior to treatment, HOKs (2 × 10^4^) were seeded and grown overnight in poly-L-lysine–coated 35 mm glass bottom dishes (MatTek Corporation) at 37°C, 5% CO_2_, then exposed to *P*. *gingivalis* 33277 (2 × 10^6^) or its vesicles (100 ng) for 0 or proper experimental times. To examine the role of Alop1 and dynasore in the bacterial invasion, the compounds were added to the medium 10 min prior to infection. The cytotoxicity of compound treatments was evaluated with a Pierce LDH Cytotoxicity Assay Kit (Thermo Scientific). There was no cytotoxicity detected under our experimental conditions.

### Confocal microscopy

HOKs were fixed with 3.8% formaldehyde in a sodium phosphate buffer at room temperature for 10 min after treatment, permeabilized with 0.1% Triton X-100 for 10 min, and blocked with 5% bovine serum albumin in PBS for 1 h. HOKs were then immunostained with pan-specific antibodies of *P*. *gingivalis* 33277, and α Tubulin or Actin mono-antibodies (Santa Cruz Biotechnology, Dallas, Texas), followed by goat anti-rabbit IgG conjugated to Alex Fluor 546 (Invitrogen). Nuclei were stained with DAPI (Invitrogen). Confocal images were acquired using a Nikon A1R confocal microscope.

For infection rate determination, the number of HOKs with intercellular *P*. *gingivalis* cells or its vesicles were determined and divided by the total number of HOKs by counting the cells in 30 random areas (5.6 μm × 5.6 μm) under the confocal microscope. Moreover, fluorescence intensity was determined in 100 individual cells using imaging software NIS-Elements AR 4.20, which reflects the level of intercellular *P*. *gingivalis* and its vesicles in the infected HOKs.

### Exit of intercellular of *P*. *gingivalis* cells assay

HOKs grown in a 6-well plate were infected with *P*. *gingivalis* 33277 cells at multiplicity of infection (MOI) of 100 for 1 h. Extracellular bacteria were removed by washing three times with PBS. The infected HOKs were then trypsinized, seeded in a glass bottom dish, and cultured with fresh media for another 20 h. To eliminate *P*. *gingivalis* cells exiting from HOKs, the growth media were supplemented with gentamicin (300 μg/ml) and metronidazole (200 μg/ml). To visualize the remaining intercellular *P*. *gingivalis*, HOKs were fixed and subjected to immunostaining and confocal microscopy.

### RNA isolation and RT-PCR

*P*. *gingivalis* was grown anaerobically in TSB in the presence or absence of Alop1 and dynasore (30 μM) for 16 h. Bacteria were harvested by centrifugation and homogenized in Trizol Reagent (Invitrogen). The RNA in the supernatant was then purified using an RNeasy mini spin column (Qiagen, Valencia, California). RNA samples were digested on the column with RNase-free DNase. Total RNA was tested using an Agilent 2100 Bioanalyzer to ensure the quality of the samples. RT-PCR analysis was performed by using an SsoAdvanced™ Universal SYBR® Green Supermix (Bio-Rad) on the CFX96 Touch™ Real-Time PCR Detection System (Bio-Rad) according to the manufacturer's instructions. Primers are listed in S1 Table. Amplification reactions consisted of a reverse transcription cycle at 42°C for 30 min, an initial activation at 95°C for 3 min, and 40 cycles of 95°C for 15 s and 60°°C for 30 s. The expression levels of the investigated genes were determined by using the formula 2^-*ΔΔ*Ct^, where *ΔΔ*CT (Cycle Threshold) = (CT_genes of test sample_−CT_16SrRNA of test sample_) − (CT_genes of control sample_ − CT_16SrRNA of control sample_).

### Statistical analyses

A student’s *t*-test was used to determine statistical significance of the differences in the invasive activities of *P*. *gingivalis* cells and vesicles in the presence or absence of Alop1 or dynasore. A *P* < 0.05 was considered significant. Values are shown ± SD unless stated otherwise.

## Results

### Inhibitory activity and efficiency of Alop1 and dynasore upon *P*. *gingivalis* invasion

**Fig 1:**
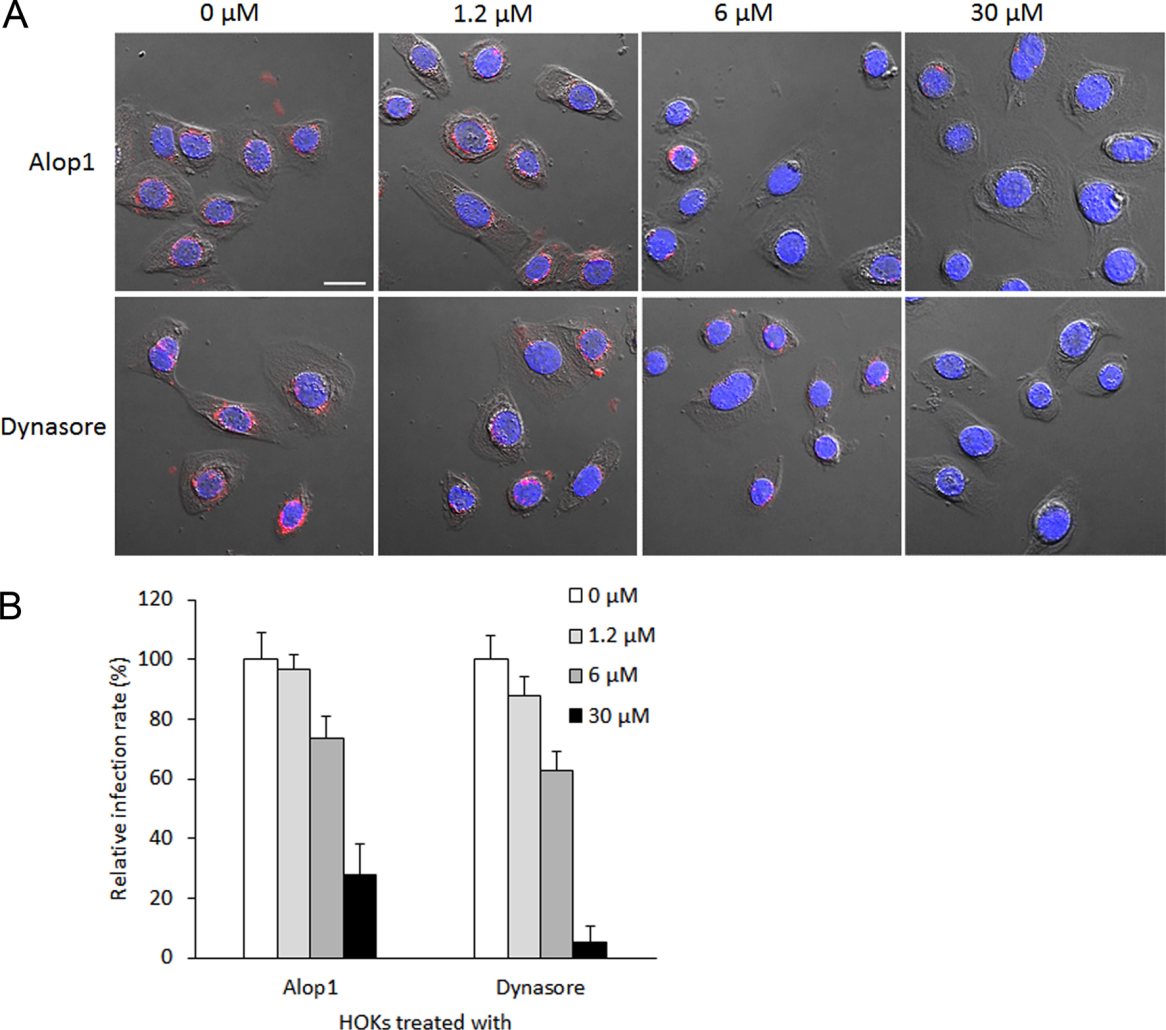
Invasive activity of *P*. *gingivalis* vesicles into HOKs in the presence of different doses of Alop1 and dynasore for 2 h. (A) *P*. *gingivalis* vesicles were stained with anti-33277 serum and a secondary antibody conjugated with Alex Fluor 546 (red), nuclei were stained with DAPI (blue), and HOKs were visualized with confocal microscopy. Scale bar, 20 μm. (B) After treatment with Alop1 or dynasore, the number of HOKs carrying intercellular *P*. *gingivalis* vesicles (infection rate) was determined by counting the infected HOKs in 30 random areas. Each bar represents the percentage of HOKs with intercellular vesicles. The SEs are indicated (*n* = 3). An asterisk indicates the statistical significance of invasive rates between *P*. *gingivalis* vesicles in the presence or absence of compounds (*P* < 0.05; *t* test).

**Fig 2:**
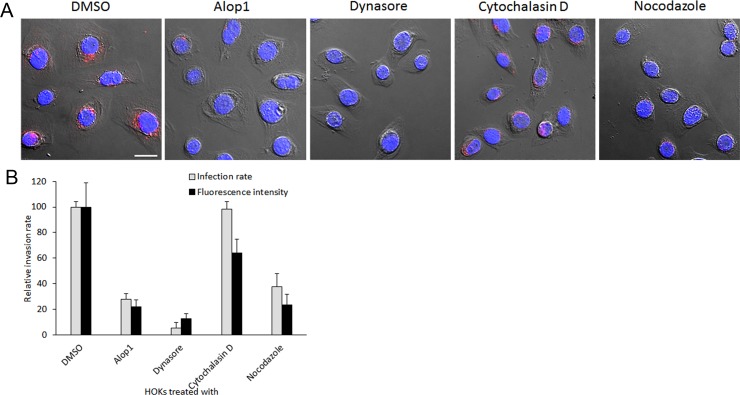
Comparison of inhibitory activities of compounds in internalization of *P*. *gingivalis* vesicles. (A) HOKs treated with 30 μM of different compounds including DMSO, Alop1, dynasore, cytochalasin D, and nocodazole for 2 h. *P*. *gingivalis* vesicles were stained with anti-33277 serum and a secondary antibody conjugated with Alex Fluor 546 (red), nuclei were stained with DAPI (blue), and HOKs were visualized with confocal microscopy. Scale bar, 20 μm. (B) Infection rate and level (average of fluorescence intensity in each cell) of HOKs treated with different compounds are presented and compared with a DMSO control. Means and SDs are indicated (*n* = 3). An asterisk indicates the statistical significance of invasive rates and levels between *P*. *gingivalis* vesicles (*P* < 0.05; *t* test).

**Fig 3:**
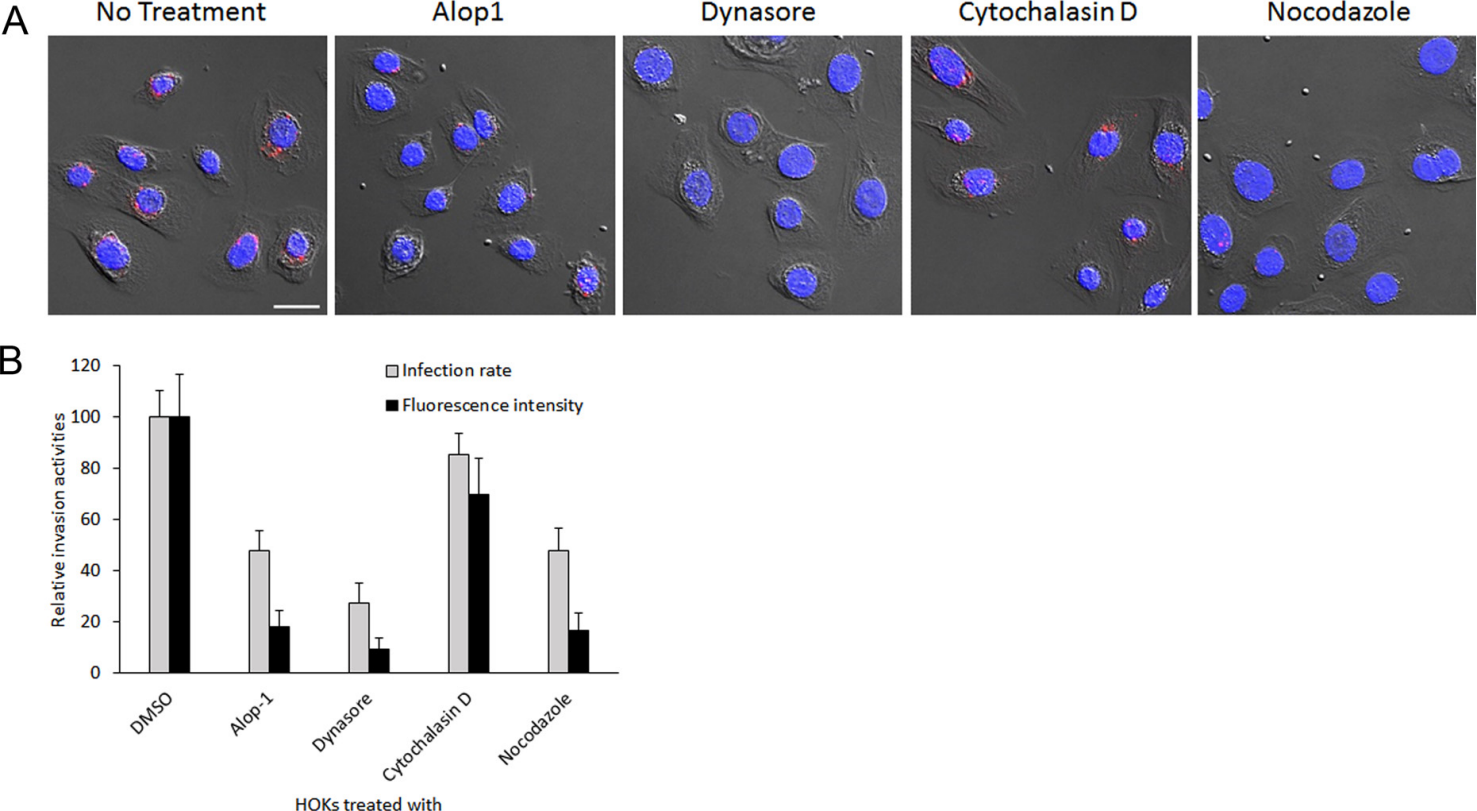
Inhibition of *P*. *gingivalis* invasion by different compounds. HOK nuclei were stained with DAPI (blue), and internalized *P*. *gingivalis* cells were stained with primary anti-33277 serum and a secondary antibody conjugated with Alex Fluor 546 (red) and visualized with confocal microscopy. Scale bars, 20 μm. (B) Each bar represents relative *P*. *gingivalis* infection rate or level of HOKs treated with compounds compared to that of untreated HOKs. An asterisk indicates the statistical significance between invasive rates or levels between *P*. *gingivalis* cells in the presence or absence of compounds (*P* < 0.05; *t* test).

Cumulating evidence has shown that both *P*. *gingivalis* and its OMVs are able to efficiently invade oral epithelial cells [8,15]. To search for compounds capable of blocking invasion of *P*. *gingivalis*, we first tested Alop1 and dynasore for their role in the bacterial vesicle invasion using confocal microscopy. Both Alop1 and dynasore displayed an inhibitory activity on *P*. *gingivalis* vesicle invasion of HOKs in a dose-dependent manner (Fig 1A). By counting 30 random areas (5.6 μm × 5.6 μm), we found that the number of HOKs with intracellular vesicles were significantly decreased in the presence of Alop1 and dynasore when compared to those observed in HOKs unexposed to the compounds. As shown in Fig 1B, Alop1 reduced the total number of HOKs with intracellular vesicles about 66% at 30 μM, and 26% at 6 μM (*P* < 0.001), and dynasore reduced the numbers by over 95% at 30 μM, and 40% at 6 μM (*P* < 0.001). Furthermore, the internalized vesicles in the HOKs were quantified by intercellular fluorescence intensity with NIS-Elements AR 4.20 imaging software. After analysis of 100 infected HOKs with or without the compound treatment (30 μM), respectively, significantly lower fluorescence intensity was detected in HOKs exposed to Alop1 or dynasore compared to that in the non-exposed HOKs (Fig 2). The effect of two well-known invasion inhibitors, cytochalasin D and nocodazole, was also tested. A similar inhibitory efficiency was found between Alop1 and nocodazole, while dynasore exhibited the highest efficiency. However, significant inhibitory activity was not observed in the presence of cytochalasin D, suggesting a microtubule-involved mechanism of inhibition. Similarly, Alop1, nocodazole, and dynasore were also found to effectively block *P*. *gingivalis* cell entry into HOKs (Fig 3A and 3B).

### Microtubule-associated *P*. *gingivalis* invasion

**Fig 4:**
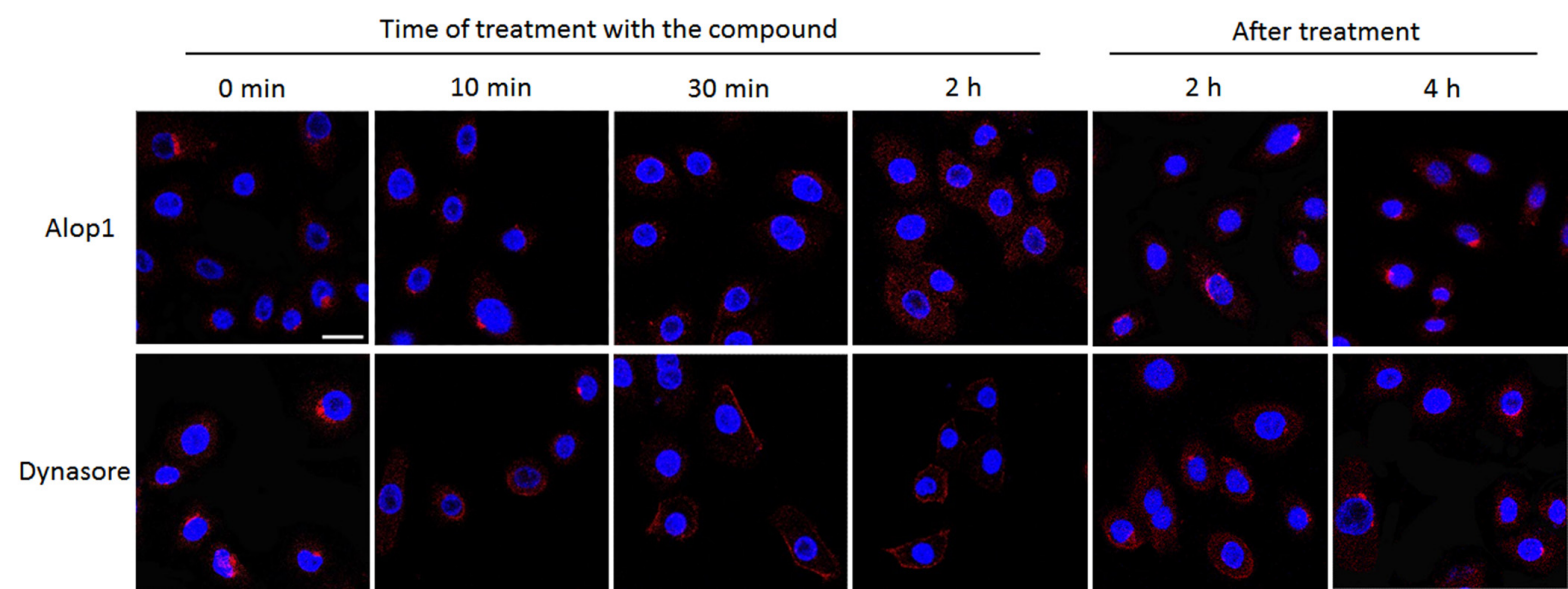
Microtubule rearrangement in HOKs induced by Alop1 and dynasore. After treated with Alop1 (30 μM) or dynasore (30 μM) for 0, 10, 30 min, or 2 h as well as recovery from treatment, HOKs were stained with anti-α-tubulin, anti-IgG with Alex Fluor 546 (red) and DAPI (blue) and visualized under a confocal microscope. Scale bar, 20 μm.

**Fig 5:**
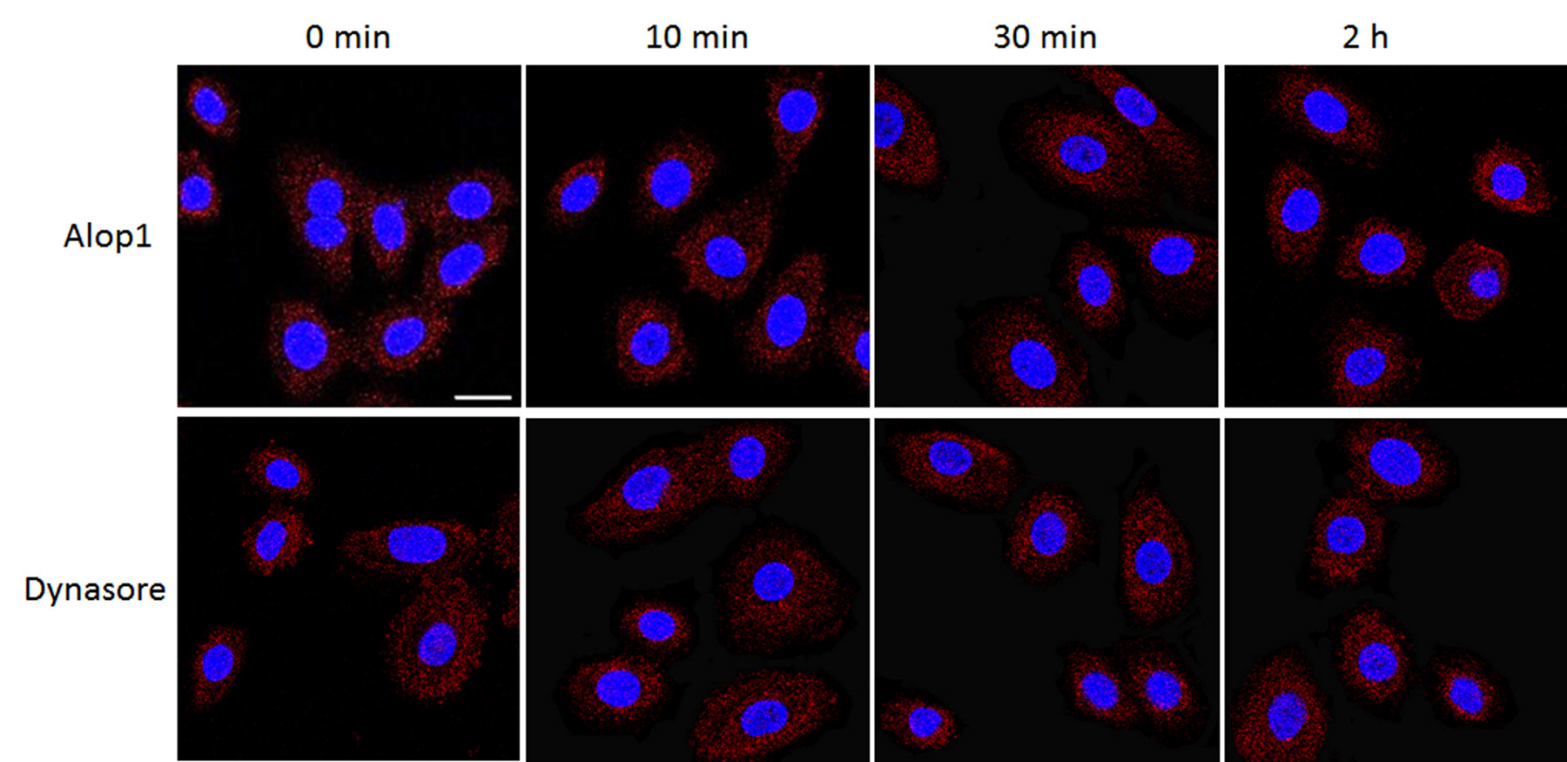
Actin arrangement in HOKs treated with compounds. After treated with Alop1 or dynasore for 0, 10, 30 min, or 2 h, HOKs were stained with anti-actin antibodies, anti-IgG with Alex Fluor 546 (red) and DAPI (blue) and visualized under a confocal microscope. Scale bar, 20 μm.

Rearrangements of host cytoskeleton have been observed during the course of bacterial infection [30]. Previous studies suggested involvement of actin filaments and microtubules in *P*. *gingivalis* invasion into epithelial cells [20,21,31]. We examined if the HOK cytoskeleton is a target of Alop1 and dynasore for inhibition of *P*. *gingivalis* invasion. Differential rearrangements of microtubules were observed in the cells treated with 30 μM Alop1 or dynasore compared to that seen in the untreated cells. Confocal microscopy revealed nucleation of microtubules near the cell nucleus in the untreated HOKs (Fig 4), consistent with previous observations [32]. Interestingly, treatment of Alop1 or dynasore each leads to a unique microtubule arrangement, which could be observed 30 min after the treatment (Fig 4). In the cells exposed to Alop1, microtubules appeared diffuse in the cytoplasm, while microtubules became more condensed and formed a cortical outer shell at the cell membrane after the HOKs were treated with dynasore. We demonstrated that the effects of Alop1 and dynasore were reversible, and recovery of differential microtubule arrangements started at 2 h after the compounds were removed from the growth media. In contrast, alteration of actin arrangement was not detected in the cells treated with Alop1 and dynasore (Fig 5), suggesting that these compounds likely target microtubule arrangement involved in invasion of *P*. *gingivalis* cells and vesicles. Since the rearrangement patterns of microtubules induced by Alop1 or dynasore were significantly distinct, mechanisms of microtubule arrangements induced by these compounds may be different.

### Dual function of dynasore

**Fig 6:**
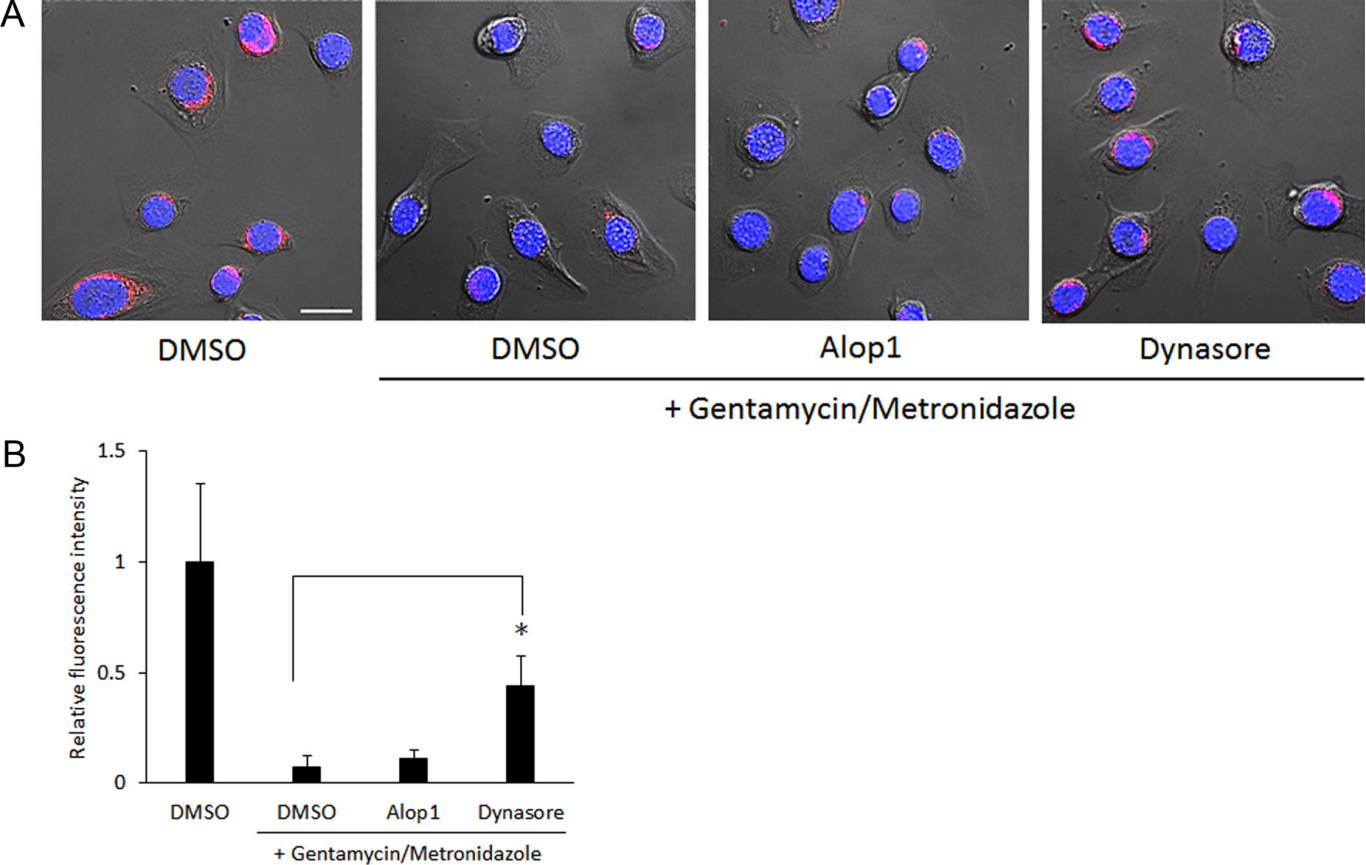
Exit of intracellular *P*. *gingivalis* cells from HOKs. (A) HOKs were cultured in the presence of antibiotics (gentamicin and metronidazole) as well as Alop1 and dynasore (30 μM) for 20 h after infection with *P*. *gingivalis* 33277. *P*. *gingivalis* cells were stained with anti-33277 serum and a secondary antibody conjugated with Alex Fluor 546 (red), nuclei were stained with DAPI (blue), and HOKs were visualized with confocal microscopy. Scale bar, 20 μm. (B) Each bar represents average of fluorescence intensity (red) in 100 infected cells cultured with Alop11 or dynasore relative to that without compounds.

Dynasore is a well-known a cell-permeable inhibitor of dynamin, a GTPase protein [26]. Therefore, we speculated that besides having an inhibitory effect on endocytosis, dynasore may also block *P*. *gingivalis* from spreading among host cells. Exit of intracellular *P*. *gingivalis* 33277 from HOKs was examined using immunofluorescence confocal microscopy. HOKs with intracellular *P*. *gingivalis* cells were cultured in the presence of 30 μM Alop1 or dynasore as well as antibiotics including gentamicin and metronidazole for 20 h. It is presumed that *P*. *gingivalis* cells exiting from HOKs would be eliminated by antibiotics, leading to a decrease in the number of intracellular bacteria. As expected, fewer *P*. *gingivalis* cells were detected in HOKs in the presence of antibiotics compared to those in HOKs not exposed to antibiotics (Fig 6A and 6B). This result indicates a cell entry, exit, and reentry cycle of *P*. *gingivalis*. Decreased intracellular *P*. *gingivalis* was also observed in HOKs treated with Alop1 and antibiotics, indicating that Alop1 did not affect the exit of *P*. *gingivalis* from HOKs. However, no significant change in the fluorescence intensity reflects the number of intracellular *P*. *gingivalis* in the presence or absence of dynasore, suggesting that dynasore may be involved in blocking intracellular bacterial exit.

### Effect of Alop1and dynasore on *P*. *gingivalis* phenotypes

**Fig 7:**
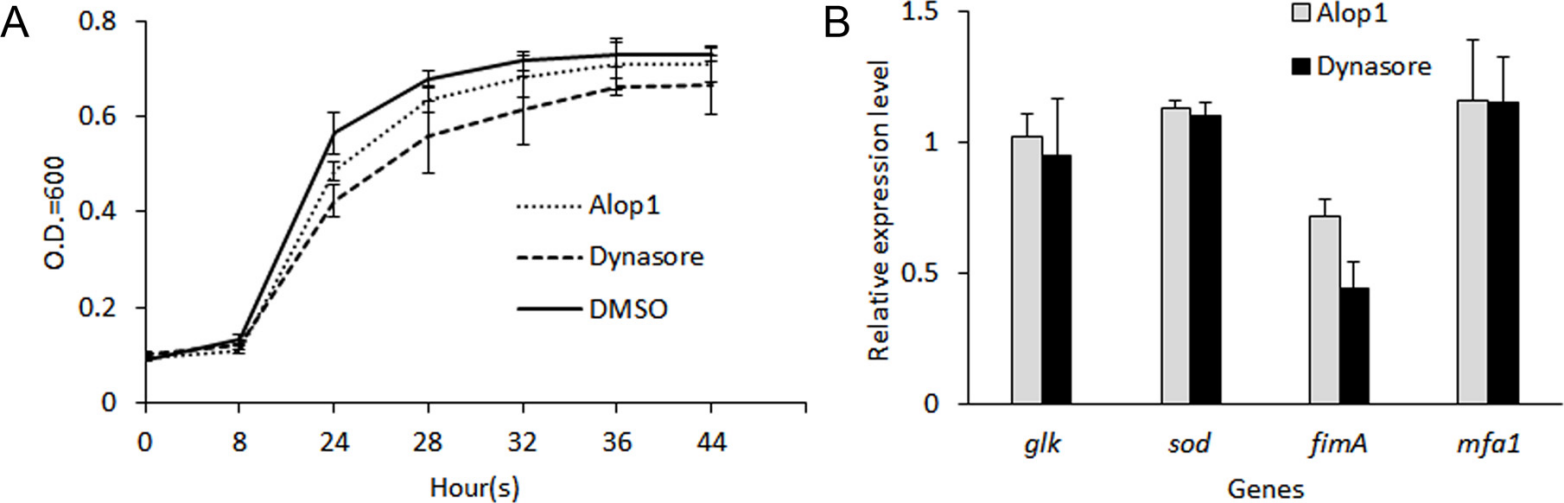
Effects of Alop1 and dynasore on bacterial growth and gene expression in *P*. *gingivalis*. (A) Comparison of the growth curves of *P*. *gingivalis* 33277 in the presence of compounds. Cells were grown in TSB media in the presence or absence of Alop1 or dynasore (30 μM). Shown in the curves are means of four samples, with error bars representing SEM. One ml aliquots were taken and the OD600 was measured over a period of 44 hr. (B) Gene expression in *P*. *gingivalis* in the presence or absence of Alop1 or dynasore (30 μM) was determined using qRT-PCR analysis. Expression levels were normalized with 16s rRNA. Representative data are shown as means with standard deviation of three biological replicates and relative to expression level of the housekeeping gene *glk*.

To determine if Alop1 and dynasore have any effect on phenotypes of *P*. *gingivalis*, we tested the bacterial growth rate and expression level of adhesins. The results showed similar patterns of the growth kinetics when *P*. *gingivalis* was cultured in TSB supplemented with or without 30 μM Alop1 or dynasore for a period of 44 h (Fig 7A). Interestingly, both Alop1 and dynasore specifically inhibited expression of *fimA*, a gene encoding a major subunit of the long fimbriae of *P*. *gingivalis* (Fig 7B). The major fimbriae are necessary for *P*. *gingivalis* attachment to oral surfaces and co-adhesion with other oral bacteria. Regulation of *fimA* expression by the compounds might be an additional mechanism to inhibit *P*. *gingivalis* entry.

## Discussion

Invasion into host cells is an important feature of opportunistic bacterial pathogens, enabling bacteria to establish pathogenic reservoirs and evade host defense mechanisms [33,34]. *P*. *gingivalis*, a nonmotile organism, utilizes invasion as a major strategy to break the oral epithelial barrier and spread in periodontal tissues. The invasion process of *P*. *gingivalis* begins with interaction between bacteria and oral epithelial cells, followed by cytoskeleton-associated internalization [15]. Effective inhibitory agents directly targeting *P*. *gingivalis* invasion have not been reported. Here we tested two small molecules, Alop-1 and dynasore, for their potential role in inhibition of *P*. *gingivalis* invasion. Our results demonstrated that both compounds inhibit *P*. *gingivalis* invasion. Dynasore is a well-known inhibitor of dynamin, essential for clathrin-coated vesicle formation and pinch-off in endocytosis [26,35,36]. Therefore, this work confirms the involvement of endocytosis in internalization of *P*. *gingivalis*, which was suggested from previous observations of the co-localization of intracellular *P*. *gingivalis* cells and an early endosome marker (EEA1) and the rearrangement of cytoskeleton using microscopic analyses [37,38]. Previously, Duncan *et al*. also suggested that *P*. *gingivalis* invasion may depend on a clathrin-mediated endocytosis based on the finding that a binding domain of gingipains interacted with clathrin of epithelial cells [39]. We further explicate that, of all the endocytic pathways, clathrin-mediated endocytosis is responsible for invasion of oral epithelial cells by *P*. *gingivalis* and its outer membrane vesicles. In contrast to dynasore, the biological mechanism(s) of Alop1 is not well studied, despite previous reports of its various biological activities [22,23,40]. The role of Alop1in microtubule arrangement revealed in this work suggests a possible mechanism of action that may account for its inhibitory activity of the bacterial entry.

Besides its role in clathrin-coated vesicle formation in endocytosis, dynamin is also involved in trafficking of these vesicles in the cells [35,41]. Therefore, we tested if dynasore can inhibit cell-cell spreading of *P*. *gingivalis* through restraining intracellular movement of clathrin-coated vesicles, since it has been suggested that entry and exit of *P*. *gingivalis* from host cells involves an endocytic recycling pathway [42]. As expected, dynasore was able to block the bacterial exit from HOKs. Dynasore’s pleiotropic effects on inhibition of *P*. *gingivalis* entering oral epithelial cells and on prevention the intracellular bacteria spreading make it a good anti–*P*. *gingivalis* candidate. The dual activities could efficiently eliminate *P*. *gingivalis* infection of oral mucosa, as super-layers constantly cast off from the epithelial surface, thus in the presence of dynasore the bacteria would not be able to reach into deep tissues.

Notably, we also observed significantly differential rearrangement of the microtubule cytoskeleton in HOKs treated with Alop1 and dynasore compared to untreated cells. The rearrangement of microtubules induced by these two compounds led to different morphologies, suggesting that Alop1 and dynasore may target distinct events of the microtubule arrangement. Unlike most invasive microbes that utilize the actin cytoskeleton for their entry into host cells, only a few bacteria, including *P*. *gingivalis*, are reported to exploit the microtubule network for their internalization [21,43]. Although our data have not provided a mechanism of Alop1 or dynasore action on microtubule, a potential link between these compounds and microtubule arrangement has been established. It was previously reported that dynamin α-tubulin and γ-tubulin are immune-precipitated with the middle domain of dynamin, which might play a role in centrosome cohesion [44]. Thus, it is reasonable to assume that dynasore, as a dynamin inhibitor, promotes centrosome splitting and prevents microtubulin nucleation in HOKs; the latter was indeed observed in this study. It should be pointed out that although dynasore is known to block the entry of several viruses including herpes simplex virus [45], it has not been considered for systemic administration as an anti-infective pharmacological agent, mainly because of the role of dynamin in broad biological functions such as neuronal transmission [41]. However, local delivery of antimicrobial agents using fiber, chip, gel, and microspheres has been recommended as a statistically and clinically significant option in the treatment of chronic periodontitis [46]. Therefore, the application of Alop1 and dynasore for elimination of *P*. *gingivalis* infection may provide an opportunity for the treatment of periodontitis.

## Supporting Information

S1 Table Oligonucleotide primers used in this study. (PDF)
