# Supplementary material for: Two Small Molecules Block Oral Epithelial Cell Invasion by Porphyromons gingivalis
Source: PLoS One. 2016 Feb 19;11(2):e0149618. doi: 10.1371/journal.pone.0149618 (PMC4760928; doi:10.1371/journal.pone.0149618)
Supplement: S1 Table — (PDF) [file pone.0149618.s001.pdf]

Table S1. Oligonucleotide primers used in this study

Gene	Primer name	Primer sequences (5'-3')	Applications
superoxide dismutase (sod)	Pg1545-235F	aattccaccacggtaagcac	For RT-PCR
	Pg1545-235R	gagccgaattgtttgtcgat	
glucose kinase (glk)	pg1737-131F	atgaatccgatccgccaccac	For RT-PCR
	Pg1737-131R	gcctcccatcccaaagcact	
mfa1	mfa1 RT-F	cagatgggttgttgctca	For RT-PCR
	mfa1 RT-R	atggaaagtgctgctggtag	
fimA	fimA1-392F	ctgtgtgtttatggcaaacttc	For RT-PCR
	fimA1-392R	aaccccgctccctgtattccga	
16s rRNA	16s-rRNA F	tgggtttaaagggtgcgtag	For RT-PCR
	16s-rRNA R	caatcggagttcctcgtgat	
